# Comparative study of the mechanical properties of woven and unidirectional fibres in discontinuous long-fibre composites

**DOI:** 10.1177/08927057221091084

**Published:** 2022-04-25

**Authors:** Réjean Belliveau, Benoit Landry, Gabriel LaPlante

**Affiliations:** 1 Département de génie mécanique, Université de Moncton, NB, Canada

**Keywords:** DLF composites, woven fibre chips, compression moulding, carbon fibre, polyetherimide matrix, composite recycling

## Abstract

Discontinuous long-fibre (DLF) composites can be made with randomly oriented unidirectional pre-impregnated composite chips. High fibre volume fraction unidirectional fibre chips provide good mechanical properties to the DLF composite architecture, which enables this material to contribute to bridging the gap between continuous-fibre and randomly-oriented short-fibre composites. However, it is well known that unidirectional fibres have highly anisotropic in-plane behaviour, which causes weak points in the parts when chips are oriented at unfavourable angles. This can be problematic since chips are randomly oriented in DLF composites. To overcome this problem, this research utilizes woven fibre chips instead of unidirectional fibre chips to fabricate DLF composites. Woven fibres diminish the potential for weak points due to their more homogenized in-plane mechanical properties. For comparison purposes, compression moulded carbon/PEI samples were made from both unidirectional chips and 5HS woven chips. Bending and tensile tests following ASTM guidelines were performed to compare both types of fibre arrangement. The results show that woven fibre chips increase the mechanical properties of the DLF composites and reduce their variability.

## Introduction

While the use of composite materials has enabled the fabrication of lighter parts, it has also increased the amount of waste generated by the industry. It is approximated that 40% of the waste generated from carbon fibre reinforced plastics come from manufacturing, and from that, more than 60% are remnants and trimmings from the processes^1^ or scraped parts. When thermoplastic resins are employed, fabricating discontinuous long-fibre (DLF) composites could be a means to minimise the CFRP waste by either using remnants from material cuts or by remoulding the scrap composites.^2^

From the effort of the 1980s to develop cost-efficient manufacturing methods for advanced composites, thermoforming emerged as a simple processing technique capable of producing parts at industrial rates, comparable to metal stamping. Aligned discontinuous-fibre composites remedied the wrinkling problems encountered when complex curved shapes were thermoformed, while maintaining the properties of continuous-fibre composites.^3^ However, the methodologies employed to produce aligned discontinuous fibres are intricate. A history of the development of the existing fibre alignment methods was presented by Such et al.^4^ A successful aligned discontinuous-fibre preform production,^5,6^ known as TuFF (Tailored Universal Feedstock for Forming), has been developed by researchers at the University of Delaware and was proven capable of translating full properties compared to its continuous carbon fibre/PEI prepreg counterpart. Such high properties for discontinuous fibres are attributable to the fact that the preform fibres are much longer than their critical length and that the fibre ends are randomly distributed in the composite.^3^ The TuFF production process can employ carbon fibres recycled by thermolysis.

**Figure 1.**
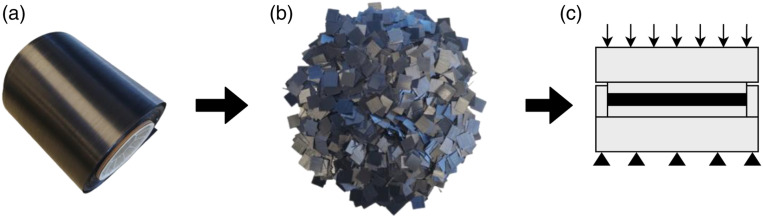
Manufacturing process of DLF composites. (a) Prepreg UD tape (b) Prepreg UD chips (c) Compression moulding process.

Newly developed compression moulding of prepreg chips is another method to produce discontinuous-fibre composites.^7,8^ Greene, Tweed and Co. (Kulpsville, PA) has marketed Xycomp DLF, a UD carbon fibre/thermoplastic system in the form of small chips. Compression moulded DLF composite research has recently generated a lot of interest in specialized industries such as aerospace and automotive. The DLF composites architecture is used to take advantage of the high fibre volume fraction ( $v_{f}$ ) provided by pre-impregnated continuous fibres, which ranges from 50–60%, while maintaining the ability to form complex shapes,^9^ thus helping to bridge the gap between continuous fibres and short fibres.^10^ DLF composites start as a continuous-fibre prepreg tape, which is cut and slit into chips as shown by Figure 1. These chips are then compression moulded into the required geometry. The chip size allows for high complexity near net-shape moulding, where parts can have varying wall thicknesses, tight radii, rib-like features, and holes.^11–12^ Therefore, a DLF composite can be a weight reducing replacement for a metal in subcomponents such as brackets.^13^ The high $v_{f}$ enhances the mechanical performance of DLF composites when compared to short fibre composites made from injection moulding. Studies show that DLF composites may have stiffnesses comparable to those of continuous-fibre quasi-isotropic laminates,^9,14,15^ but their strength is significantly lower.

Early studies suggested that in-plane isotropy can be achieved in DLF composites.^9^ However, recent studies have shown that when chip flow is encountered during moulding, in-plane isotropic properties can no longer be assumed.^16–18^ Chip flow leads to fibre alignment in the direction of flow, creating a highly anisotropic material where lower properties are encountered perpendicular to the flow. Furthermore, using digital imaging correlation, some researchers have observed highly nonuniform strain fields caused by variability in fibre orientation.^14,19^ Weak points in the material due to unpreferred chip orientations is a source of serious concern in load bearing components. Woven continuous fibres have the potential to alleviate this problem and lead to a more uniform in-plane behaviour.

So far, research has predominantly been focused on DLF composites fabricated with UD chips, yet UD fibres are not the only type of fibres used in the industry; woven fibres are highly used. While UD chips are currently being manufactured as virgin material, there is also great potential for using chips that are cut from remnants or scrap thermoplastic parts that would otherwise go to landfills. These chips could be made from both UD and woven materials, without costly fibre extraction methods.

The fabrication of DLF composites may have its challenges. Abdul^20^ has analyzed the moulding of semi-impregnated woven fibres. His study showed that fibre wetting was difficult where dry fibres blocked the flow of chips, causing defects in the part. Higher dwell times as well as higher mould squeeze rates reduced the jamming of chips. Chip jamming is not the only concern during moulding; Landry et al.^21^ demonstrated that a shrinkage related pressure loss during moulding can cause significant surface defects in a semi-crystalline (PEEK) matrix composite, resulting in mechanical performance reduction.

This paper presents an investigation into the use of woven fibres as a means to increase the performance of DLF composites. The main objectives of this work are to demonstrate that woven fibre chips can be used in DLF composites without any major alterations to the fabrication method, and to analyze the effect of using woven fibre chips on the mechanical properties when comparing to the conventional UD chips. The failure modes of these types of composites will also be observed.

## Experimental procedures

### Material

The material used in this study was Toray TC1000, a thermoplastic carbon fibre composite using polyetherimide (PEI) as its matrix component. The glass transition temperature ( $T_{g}$ ) of PEI is 217°C and its molecular structure is amorphous. Unidirectional (UD) prepreg tape and 5HS woven pre-consolidated composites are compared in this study. The composite systems were selected based on having properties and $v_{f}$ as similar as possible, with the constraint of commercial availability. The selected UD tape is made from AS4 carbon fibres^22^ while the woven composite uses the standard modulus FT300 B fibres.^23^ Both types of fibres have similar stiffnesses of approximately 230 GPa. However, AS4 fibres are significantly more resistant, having a tensile strength of 4237 MPa compared to 3530 MPa for the FT300 B fibres. The UD tape has a thickness of 0.14 mm and a $v_{f}$ of 59% while the pre-consolidated woven composite has a thickness of 0.26 mm and a $v_{f}$ of 50%. Additional information on these materials can be found in the manufacturer datasheet.^24^ DLF chips were cut from the UD tape and pre-consolidated composite into chips of 12.7 mm × 12.7 mm using a hydraulic sheet metal sheering press. Square chips were used to eliminate the possibility of variability in the properties caused by the aspect ratio of the chips.

### Equipment

**Figure 2.**
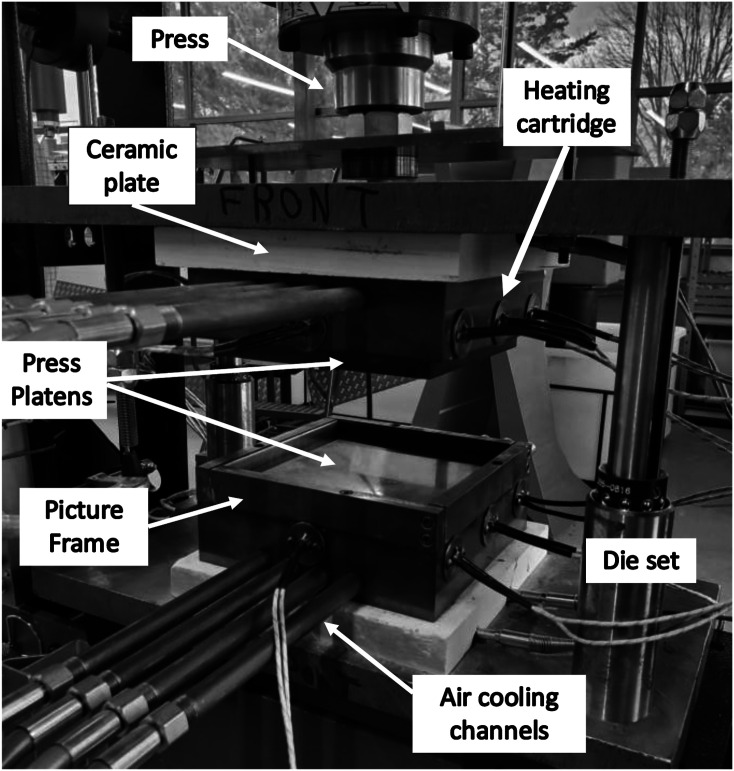
Flat panel moulding setup.

A compression moulding technique was used to fabricate the panels from which samples were cut. A picture frame style purpose-built mould was fabricated as shown in Figure 2. This type of mould was previously used for moulding DLF composites.^17,21^ It is made from P20 tool steel which has been treated to prevent part adhesion. The treatment consists of initially cleaning the mould using Chem-Trend Zyvax FreshStart mould cleaner, then applying Chem-Trend Zyvax Sealer and finally applying Chem-Trend Zyvax CompositeShield release agent.^25^ The moulding surface is 152 mm × 152 mm and the maximum panel thickness is 5 mm . A total of 16 Watlow heating cartridges (250 W each) are integrated in the mould. The heating cartridges are controlled via four PID loops, two for each platen, creating two control zones on each platen. The mould is cooled by eight air channels passing through it. Components of the mould are shown in Figure 2. The platens are insulated with 25 mm thick ceramic plates. A maximum temperature difference of 8°C was observed between the centre and a corner of the mould during cooling from 320°C. A 30 US ton press was used to compress both platens and create pressure on the material.

### Panel fabrication

**Figure 3.**
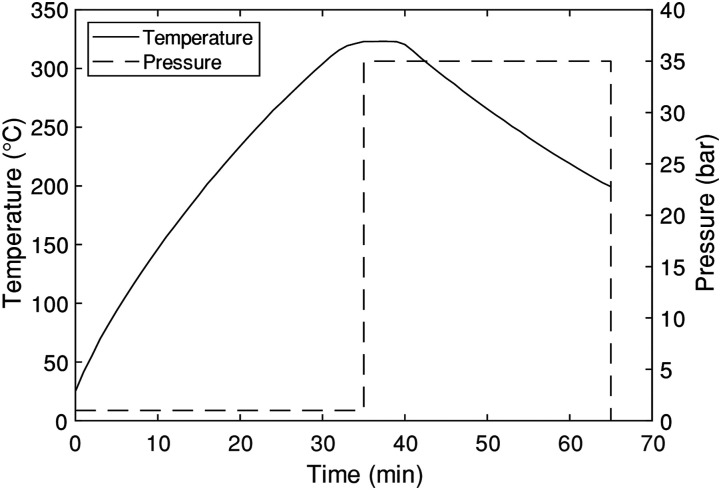
Moulding cycle used for panel fabrication. The temperature shown was measured by an embedded thermocouple located at the center of the moulding surface.

Panels made from continuous fibres and from DLF chips were moulded with the same process parameters. The process temperature was set at 320°C, as suggested by the manufacturer,^24^ with a dwell time of 5 min. When the process temperature was reached, a pressure of 35 bar was applied, based on the study by Hou et al.^26,27^ A debulk pressure cycle was also used at the beginning of the dwell to promote good material consolidation, as suggested by the manufacturer.^28^ After the dwell, compressed air was supplied to the cooling channels and the temperature was lowered to 200°C for the demoulding of the panels. When moulding multiple panels, the material for the next part was added at 200°C to reduce the processing time. The temperature at the centre of the mould and the pressure exerted on the mould during the fabrication cycle are summarized in Figure 3.

**Figure 4.**
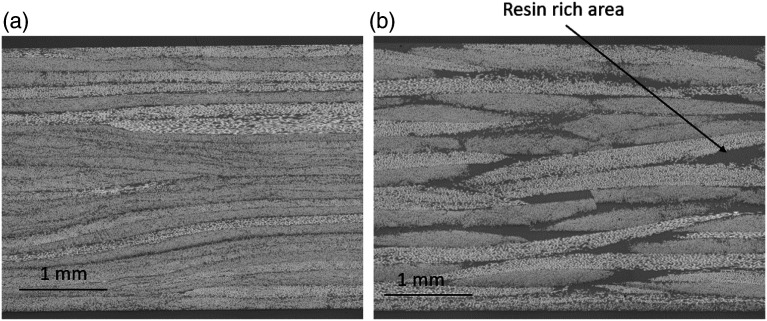
DLF panel micrographs show the defect free material (a) DLF composite with UD chips (b) DLF composite with woven chips. The dark gray areas in (b) are resin-rich areas.

To fabricate the reference continuous-fibre panels, 152 mm × 152 mm plies were cut from the roll of material and laid up in the mould following the stack up sequence given in Table 1. In total, the UD and woven panels were composed of 21 and 12 plies, respectively. To fabricate the DLF panels, weighed quantities of material were placed in the mould: 112 g of UD chips and 105 g of woven chips were used for the UD and the woven DLF plates, respectively. Since no material was lost during fabrication, $v_{f}$ of the plate was assumed to be the same as in the raw material. To verify the quality of the material, four cross-section micrographs at 100× magnitude were taken on each of the fabricated panels. Typical micrographs are shown in Figure 4. These micrographs show that the DLF panels are porosity free, which validates the moulding cycle of Figure 3. The surface finish was also defect free, which reveals an advantage of PEI (amorphous) over PEEK (semi-crystalline)^16^ as the matrix for compression moulding applications.

**Table 1.** Summary of the mechanical test performed.

Type of panel	Test	Standard	No. of specimen
DLF UD chips	Tensile	ASTM D3036	10
DLF woven chips	Tensile	ASTM D3036	10
Continuous UD ${\left[{\left(90{}^{\circ}/45{}^{\circ}/-45{}^{\circ}/0{}^{\circ}\right)}_{4}\right]}_{s}$	Tensile	ASTM D3036	5
DLF UD chips	Bending	ASTM D7264	10
DLF woven chips	Bending	ASTM D7264	10
Continuous UD ${\left[{\left(0/90\right)}_{5}/\bar{0}\right]}_{s}\;\;\;\;$	Bending	ASTM D7264	5
Continuous UD ${\left[{\left(90/0\right)}_{5}/\bar{90}\right]}_{s}\;\;\;\;$	Bending	ASTM D7264	5
Continuous woven ${\left[0/90\right]}_{3s}$	Bending	ASTM D7264	5

### Mechanical testing

#### Tensile tests

Tensile testing was performed according to the ASTM D3039 standard^29^ on specimens cut from the fabricated plates. To eliminate the possibility of having specimens with chip orientation being affected by the edges of the mould, as observed by Léger et al.,^17^ a strip of 12.7 mm was removed along the edges of the panels. Due to the mould dimension limitations, a specimen length of 152 mm and width of 25 mm was used, giving a length of 102 mm between the grips of the testing machine. Sandpaper (100 grit) was used for tabs on the specimen to prevent slippage within the grips. Strain was measured using an extensometer equipped with an extended gauge length (50 mm). Stress and stiffness calculations for the tensile tests followed the ASTM standard. Stress and strain measurements were repeated four times (different locations on the specimen for each measurement) and an average stiffness based on the four measurements was obtained for each specimen, as recommended by Feraboli et al.^14^ The test was carried to the ultimate load on the last repetition.

#### Bending tests

Bend testing was performed according to the ASTM D7264 standard, procedure B (four-point bending).^30^ A span of 96 mm was selected for the 3 mm nominal specimen thickness, resulting in a span-to-thickness ratio of 32. The fabrication process led to a coefficient of variation (CoV) of only 1.9% in the thickness of the specimens. A specimen width of 25 mm was chosen based on the 12.7 mm × 12.7 mm chip dimensions (i.e. the specimen width is twice that of the chips dimensions). For the same reason as in the tensile testing, a 12.7 mm strip on all edges of the panel was discarded. Flexural chord modulus calculations followed the ASTM standard. The strain range was 0.2% with the starting point at 0.1% and end point at 0.3%.

A summary of the mechanical test performed in this study is shown in Table 1. To further study the difference in performance between the UD and woven architectures and to establish baselines for these configurations, continuous-fibre quasi-isotropic and cross-ply composite panels were also fabricated and tested (see Table 1). The continuous UD fibre composite used for tensile testing is a quasi-isotropic laminate that was tested to obtain a reference to compare the in-plane strength and modulus of the DLF composite, which is theoretically quasi-isotropic for a random distribution of chip orientation. The UD cross-ply stack-up sequences were selected to show the effect of the outer ply during bending.

The comparison between DLF and continuous-fibre composites properties can be expressed in the form of a knockdown factor $K_{f}$ given as

(1) $$K_{f}=\frac{X_{i}^{DLF}}{X_{i}^{CF}}$$

where $X_{i}^{DLF}$ is the value of a mechanical property for a DLF composite and $X_{i}^{CF}$ is the value of the same property for a reference continuous-fibre composite. Index i stands for either W (woven chips) or UD (unidirectional) fibres. Factor $K_{f}$ is therefore quantifying the performance loss when converting the continuous fibres into discontinuous randomly oriented fibres. The references utilized in this study are the quasi-isotropic laminate for the tensile test results and the cross-ply laminate ${\left[{\left(0/90\right)}_{5}/\bar{0}\right]}_{s}\;$ for the bending test results.

## Results

### Tensile tests

**Figure 5.**
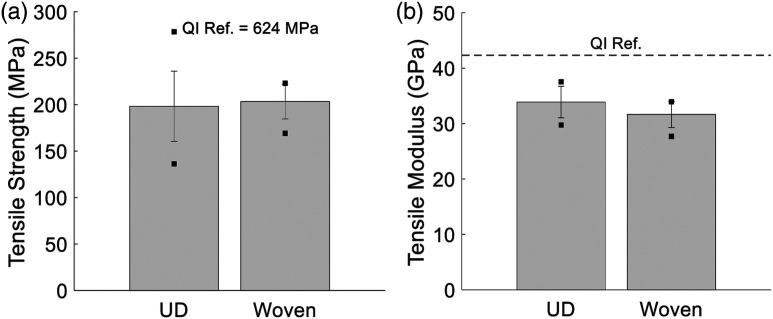
Mean tensile properties for DLF composite with UD chips and woven chips. Error bars and dots represent the standard variation and the maximum and minimum values, respectively. (a) Strength measurements (*n* = 20) (b) Modulus measurements (*n* = 20). Quasi-isotropic (QI) reference is based on a UD continuous-fibre composite with the following layup ${\left[{\left(90{}^{\circ}/45{}^{\circ}/-45{}^{\circ}/0{}^{\circ}\right)}_{4}\right]}_{s}$ .

Mean strength and modulus results from tensile tests for both UD and woven DLF panels are shown in Figure 5, where the error bars represent the standard deviation, and the dots represent the maximum and minimum values. To compare both materials, the analysis of variance (ANOVA) method was used, with a significance level of 0.05 and testing the null hypothesis that there is no difference in means against the alternative that the means are different. Based on the results shown in Figure 5, the strength of both materials is statistically equal with a *p*-value of 0.71. However, UD chip specimens show a much higher coefficient of variation (CoV) than woven chip specimens, with values of 19% and 9%, respectively. The maximum strength achieved in specimens made from UD chips is significantly higher than the one obtained from woven chips. However, the minimum value obtained from the woven chips is significantly higher than that of the UD chips. This is of utmost importance as design is typically driven by minimum material properties. It is also worthwhile to note that while the mean strength values of both DLF materials are the same, the $v_{f}$ for the woven fibres used is 9% lower than that of the UD fibres. In addition, the FT300 B fibres used in the woven chips are significantly less resistant than the AS4 fibres used in the UD chips. This implies that the strength-to- $v_{f}$ ratio is higher with the woven chips. Stiffness measurements also show statistically similar results between both materials, with a *p*-value of 0.08, even though $v_{f}$ is 9% lower in woven chips and the fibre stiffness is similar in both materials. This suggests that the higher heterogeneity of the UD chips have a greater impact on the stiffness of the specimen then the lower $v_{f}$ of the woven material.

Using the UD quasi-isotropic laminate as the reference for both types of chips, low strength *K*_*f*_’s of 0.32 and 0.33 and respectable modulus *K*_*f*_’s of 0.79 and 0.74 are yielded by the UD and woven chip composites, respectively. This agrees with the observations of Feraboli et al.^9,14^ These *K*_*f*_’s are significantly lower than those of composites using TuFF preforms (aligned discontinuous fibres). This may be explained by the fact that, in one chip, all fibre ends coincide, which affects load transfer within DLF composites.

### Bending tests

**Figure 6.**
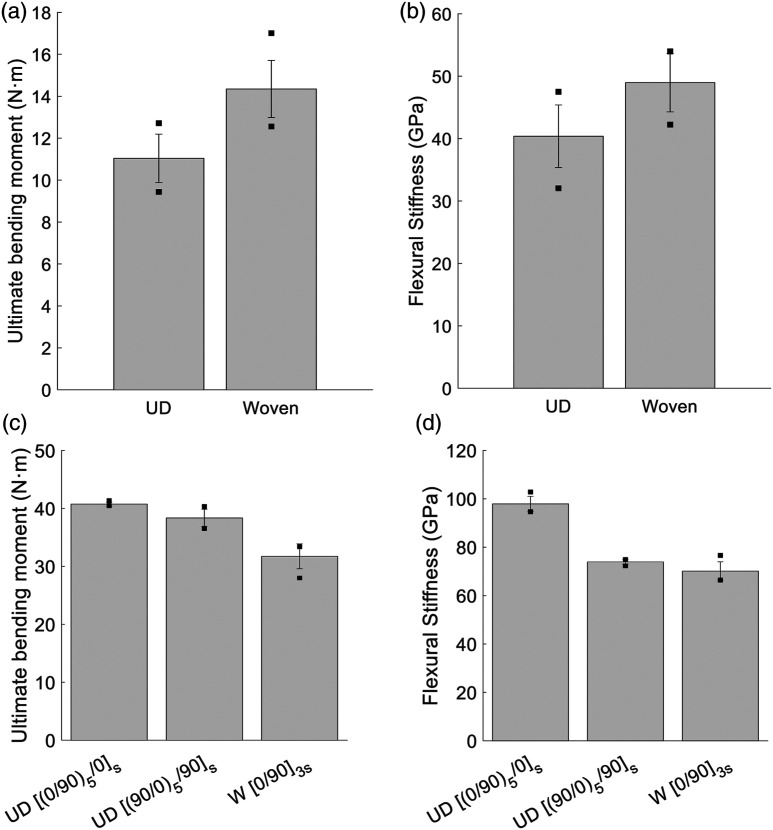
Mean ultimate bending moment and flexural stiffness. (a) Ultimate bending moment for DLF composite (b) Flexural stiffness for DLF composite (c) Ultimate bending moment for continuous-fibre composites (d) Flexural stiffness for continuous-fibre composites. The error bars and dots represent the standard deviation and the maximum and minimum values, respectively (*n* = 35).

Mean values for the ultimate bending moment and the flexural modulus are shown in Figure 6. Ultimate bending moments are compared instead of stresses because of the random orientation of chips inside of the DLF composites. The variation in thicknesses (CoV of 1.9%) was deemed not significant enough to be a factor when comparing the ultimate bending moment between specimens. Figures 6(a)-(b) and (c)-(d) present the results from the DLF panels and the continuous-fibre panels, respectively. Error bars and dots have the same meaning as in Figure 5. In Figures 6(a) and (b), the results show increases of 29% in the ultimate bending moment and 20% in the flexural stiffness when woven fibre chips are used instead of UD chips for DLF composites. The increases are statistically significant in both strength and stiffness, with *p*-values significantly lower then the 0.05 threshold.

Here, for discussion purposes, the knockdown factors will be based on UD continuous fibres for UD DLF composites and based on woven continuous fibres for woven DLF composites. Using UD cross-ply ${\left[{\left(0/90\right)}_{5}/\bar{0}\right]}_{s}$ as the reference, the UD fibre DLF has an ultimate bending moment $K_{f}$ of 0.27. On the other hand, using woven cross-ply laminate ${\left(0{}^{\circ}/90{}^{\circ}\right)}_{3s}$ as the reference, the woven fibre DLF has a $K_{f}$ of 0.45. This indicates that woven fibre DLF outperforms the UD DLF composite in translating the properties of its continuous-fibre analogous architecture. It is also the case for the stiffness, where *K*_*f*_’s of 0.41 and 0.69 are found for UD and woven DLF’s, respectively. In addition, based on continuous-fibre results (Figures 6(c) and (d)), UD fibres have a better mechanical performance than woven fibres; however, in DLF composites, it is not the case.

## Discussion

### Mechanical performance

Strength and stiffness in fibre-reinforced plastics are highly dependent on the fibre direction. Therefore, the high variability of the tensile test results with the UD chip specimens is suggested to be caused by chip angle. When a high number of chips are oriented in the direction of the load, the strength and stiffness increase. However, the performance of the composite is greatly diminished when most of the fibres are oriented at high angles (i.e. close to 90° from the load direction), causing weak spots in the part. Since the UD chips have a more predominant weakness than the woven fibres, the variability of the composite properties increases. Woven fabrics have an advantage because their weakness, which is when the chip is oriented at 45° to the load, is far less predominant. Therefore, in this study, even though $v_{f}$ and fibre tensile strength were lower in the woven chip DLF composite, a performance increase was observed.

For tensile testing, UD chips did not show a significant decrease in mean performance when compared to woven chips, since the load is evenly distributed across the entire cross section of the specimen in this case. However, when the specimens are subjected to bending loads, the chips located at both surfaces of the specimen are subjected to the highest strain. Therefore, if most of the UD chip fibres near the surface of the specimen are unfavourably orientated (i.e. high angle), the bending strength and stiffness will be reduced. This is suspected to be the case for the bending tests results shown in Figure 6, where woven chips yielded a statistically higher strength and stiffness, while keeping a low variability. This assumption is based on the continuous-fibre results (Figures 6(c) and (d)), which show that the ply nearest to the surface has a significant impact on the mechanical performance in bending. In general, UD chips lead to a more significant mechanical performance loss when compared to the woven chips.

It is noteworthy to draw attention to the fact that while the tensile strength of the UD chip and the woven chip specimens are almost equivalent (Figure 5(a)), the ultimate bending moment of the woven chip specimens is significantly higher than that of its UD counterpart. This suggests that the compressive properties of the woven DLF composites are superior to those of the UD DLF composites. This will be discussed further in the microstructure analysis section.

### Microstructure analysis

**Figure 7.**
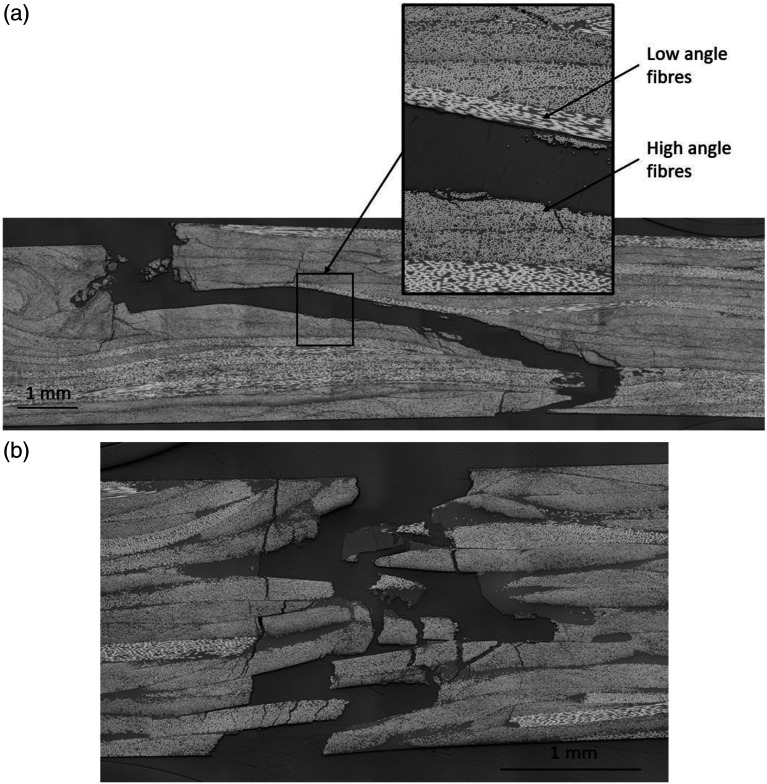
Typical micrographs of DLF composite specimens tensile test failure (*n* = 8) (a) UD chips (b) Woven chips.

Micrographic analysis revealed interesting differences between the failure modes of each type of chips. Cross-section micrographs were taken on edges of both specimen types. Figure 7 shows typical micrographs of the tensile test failures for both cases. The crack in the UD chip specimen starts at the surface at a location where chips are oriented transversally to the load (i.e. high fibre angles). The crack then follows a path at the interface between two chips (plies) that have a large difference in orientation, presumably due to high shear stresses between the adjacent chips. This was verified by measuring the orientation of the two adjacent chips with the ellipse method described by Belliveau et al.^16^ Fibres at 0° are defined as being parallel to the longitudinal axis of the specimen. Analysis of the chip orientation on each side of the crack found in Figure 7(a) revealed an original contact between low fibre angles of approximately 9° and high fibre angles of approximately 81°, resulting in an angular difference of 72°. Similar results were observed in all the other micrographs (n=4) of UD chip specimens, where a mean angular difference of 68° with a CoV of 6% was measured at the cracked interface between chips. It is supposed that the difference in orientation between adjacent UD chips caused the delamination. By contrast, DLF specimens made from woven fibre cracked in a much straighter line, in the through-thickness direction of the specimen, as shown in Figure 7(b). Woven chips clearly lead to a more random failure mode, which was characterized by chip breakage and limited delamination.

**Figure 8.**
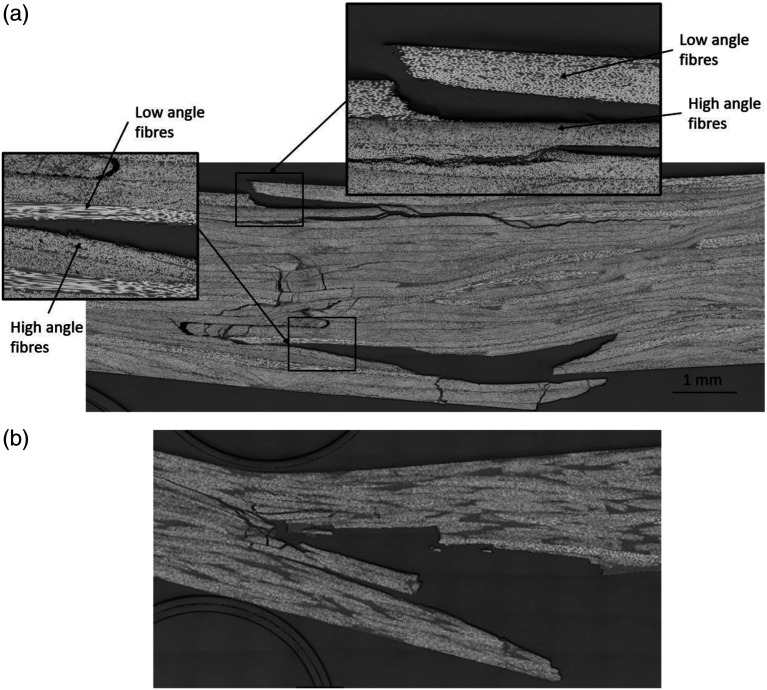
Typical micrographs of DLF composites specimens bending test failure. (a) UD chips (b) Woven chips.

Failure modes in bending show similar results, as shown in Figure 8. UD chips produce delamination (Figure 8(a)), and it occurs in tension (bottom surface) as well as in compression (top surface). It is unknown which crack initiated first but it appears that the crack on the tension side progressed deeper within the specimen by either transverse cracking through high fibre angle chips, delamination along chip interfaces when low fibre angle chips are encountered, or through-thickness cracking at the end of chips. Failure initiation (at both outer surfaces) appears in areas where high angle fibres are located, indicating that matrix failure occurred. Delamination in bending is also located at the interface between high and low fibre angle chips, similar to the results obtained in tensile tests. Using the same method to extract fibre angles, the mean angular difference calculated is 70° with a CoV of 5%. It is to be noted that UD DLF composites have a higher number of chip interaction due to the greater number of (thinner) chips across the thickness of the composite. Therefore, having chip interactions with a large angle difference is more likely than in the woven chips composite.

The micrograph of the woven chip DLF composite fracture (Figure 8(b)) reveals that fracture initiated at the tensile surface (bottom) of the specimen and progressed inward, while the compression surface (top) did not crack. Since the two DLF composites have approximately the same average tensile strength (Figure 5(a)), the higher bending strength of the woven chip DLF composite may be attributed to its better performance in compression compared to the UD chip DLF composite.

**Figure 9.**
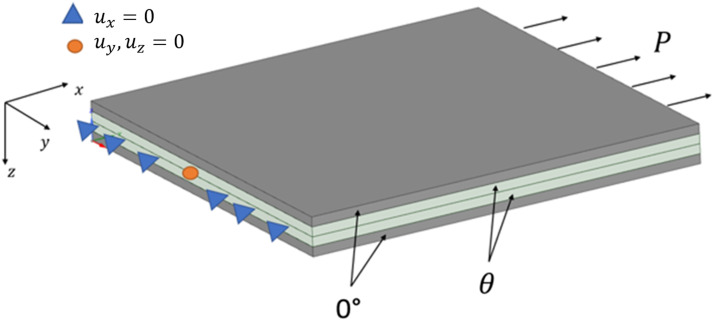
Boundary conditions used in the finite element model.

To explain why delamination is occurring at the interface between adjacent chips with large differences in fibre angles, a finite element model was developed to extract the shear stress between chips (Figure 9). The model represents a 4-ply composite with the following stack up sequence ${\left[0{}^{\circ}/\theta \right]}_{s}$ . Contact elements with bonded behaviour were introduced at the interface between the plies. The plate size was 12.5 mm × 12.5 mm and the ply thickness was the same as the respective physical materials. Material properties used for this model are shown in Table 2. The woven properties were based on the manufacturer’s datasheet.^19^ The UD properties were measured experimentally following the ASTM D3039 standard.^29^ The boundary conditions were defined to represent a unidirectional tensile test where one end is fixed, and a distributed load (P) is applied to the opposing end, as shown in Figure 9.

**Table 2.** Material properties used in the finite element model.

	UD	Woven
E_1_ (GPa)	120	56
E_2_ (GPa)	9	57
G_12_ (GPa)	5.2	4.5
V_12_	0.29	0.05

**Figure 10.**
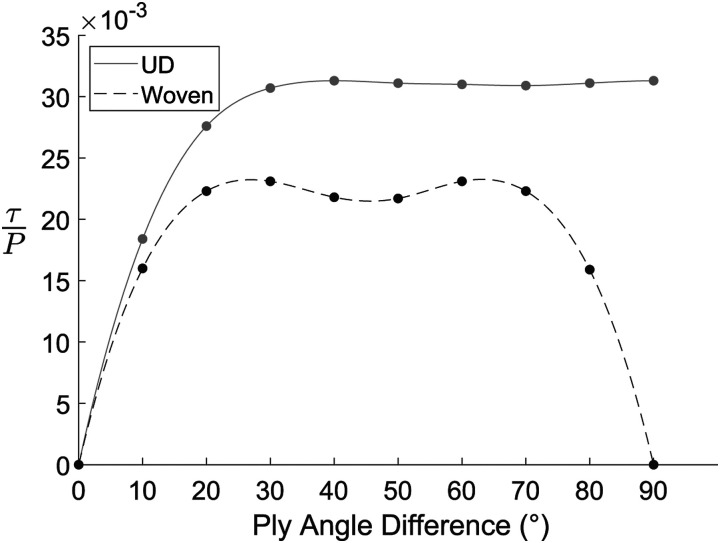
Tensile loading induced maximum shear stress at the interface between plies with angle difference ( θ ).

The normalized maximum shear stress (τ/P) between the 0° ply and the ply angled at θ is shown in Figure 10, where the angle θ varies between 0° and 90°. Even at low angle differences (i.e. 30°) the shear stress between the UD material plies is already 32.9% higher than between the woven material plies. Due to the configuration, the maximum interfacial shear stress has a symmetric pattern about the 45° angular difference for the woven material while the interfacial shear stress in the UD material remains high up to 90°. Therefore, it is inferred that UD chips stacked on top of each other with random fibre orientations are far more susceptible to cause high interfacial shear stresses compared to woven chips. The high shear stress between chips is suspected to be the cause for the delamination observed previously.

### Moulding woven DLF composites

The compression moulding of woven DLF composites with pre-consolidated fibres was successful. The mould was filled evenly, and no part defect was observed. The exact same procedure was followed for moulding with UD chips and woven chips, and the moulding of pre-consolidated woven chips did not cause any problems such as jamming. This implies that pre-consolidated woven chips are adequate to be used in DLF composites, thus increasing the potential of reusing/remoulding trimmings and scraps from thermoplastic matrix composites.

Using PEI (which has an amorphous structure) as a matrix required significantly less pressure to mould defect-free flat panels when compared to studies where PEEK (i.e. semi-crystalline structure) was used as the matrix.^2,17,21,31,32^ The loss in pressure observed in studies using PEEK was caused by the crystallization of the material upon cooling, which created a sudden shrinkage in the material, thus causing porosity. This phenomenon was not observed with the PEI matrix. In addition to the reduction in pressure requirement, the moulding temperature of PEI is also significantly lower than that of PEEK, resulting in the possibility of faster cycle times. This suggests that matrices with amorphous molecular structures are more suitable for compression moulded DLF composites.

## Conclusion

A new approach to the fabrication of discontinuous long-fibre composite has been studied, where woven fibre chips are utilized instead of UD fibre chips. Based on the continuous-fibre composite properties, it was hypothesized that mechanical properties would increase with the use of woven fibres in DLF composites. Tensile and bending tests results support this hypothesis; the mean strength in tension and bending as well as the flexural stiffness were shown to be higher with woven chips. The woven DLF composite outperformed the UD DLF composite, even though the woven chips employed had a lower fibre volume fraction and a lower fibre tensile strength than its UD counterpart. It was also shown from the tensile strength measurements that the coefficient of variation is significantly lower with woven chips compared to UD chips. This suggests that the more uniform in-plane properties of the woven chips lead to higher mechanical properties in woven DLF composites. The failure mode observed in the UD DLF composites included delamination and matrix failure, whereas the failure mode in the woven chips DLF composite showed fibre failure, i.e. complete chip failure.

Up to now, pressure moulded DLF composites have essentially been performed with UD chips, consequently limiting the potential for the recycling of composites. This study has shown that moulding DLF composites from woven chips is possible without any alteration to the fabrication process and suggests that there is a mechanical advantage to this new approach.
